# Ultrasound image analysis using deep neural networks for discriminating between benign and malignant ovarian tumors: comparison with expert subjective assessment

**DOI:** 10.1002/uog.23530

**Published:** 2021-01-02

**Authors:** F. Christiansen, E. L. Epstein, E. Smedberg, M. Åkerlund, K. Smith, E. Epstein

**Affiliations:** ^1^ School of Engineering Sciences, KTH Royal Institute of Technology Stockholm Sweden; ^2^ Department of Clinical Science and Education, Karolinska Institutet, and Department of Obstetrics and Gynecology, Södersjukhuset Stockholm Sweden; ^3^ Harvard Extension School Harvard University Cambridge MA USA; ^4^ Science for Life Laboratory, School of Electrical Engineering and Computer Science KTH Royal Institute of Technology Stockholm Sweden

**Keywords:** classification, computer‐aided diagnosis, deep learning, machine learning, ovarian neoplasm, ovarian tumor, transfer learning, ultrasonography

## Abstract

**Objectives:**

To develop and test the performance of computerized ultrasound image analysis using deep neural networks (DNNs) in discriminating between benign and malignant ovarian tumors and to compare its diagnostic accuracy with that of subjective assessment (SA) by an ultrasound expert.

**Methods:**

We included 3077 (grayscale, *n* = 1927; power Doppler, *n* = 1150) ultrasound images from 758 women with ovarian tumors, who were classified prospectively by expert ultrasound examiners according to IOTA (International Ovarian Tumor Analysis) terms and definitions. Histological outcome from surgery (*n* = 634) or long‐term (≥ 3 years) follow‐up (*n* = 124) served as the gold standard. The dataset was split into a training set (*n* = 508; 314 benign and 194 malignant), a validation set (*n* = 100; 60 benign and 40 malignant) and a test set (*n* = 150; 75 benign and 75 malignant). We used transfer learning on three pre‐trained DNNs: VGG16, ResNet50 and MobileNet. Each model was trained, and the outputs calibrated, using temperature scaling. An ensemble of the three models was then used to estimate the probability of malignancy based on all images from a given case. The DNN ensemble classified the tumors as benign or malignant (Ovry‐Dx1 model); or as benign, inconclusive or malignant (Ovry‐Dx2 model). The diagnostic performance of the DNN models, in terms of sensitivity and specificity, was compared to that of SA for classifying ovarian tumors in the test set.

**Results:**

At a sensitivity of 96.0%, Ovry‐Dx1 had a specificity similar to that of SA (86.7% *vs* 88.0%; *P* = 1.0). Ovry‐Dx2 had a sensitivity of 97.1% and a specificity of 93.7%, when designating 12.7% of the lesions as inconclusive. By complimenting Ovry‐Dx2 with SA in inconclusive cases, the overall sensitivity (96.0%) and specificity (89.3%) were not significantly different from using SA in all cases (*P* = 1.0).

**Conclusion:**

Ultrasound image analysis using DNNs can predict ovarian malignancy with a diagnostic accuracy comparable to that of human expert examiners, indicating that these models may have a role in the triage of women with an ovarian tumor. © 2020 The Authors. *Ultrasound in Obstetrics & Gynecology* published by John Wiley & Sons Ltd on behalf of International Society of Ultrasound in Obstetrics and Gynecology.

## Abstract 2

This article's abstract has been translated into Spanish and Chinese. Follow the links from the abstract to view the translations.

## RESUMEN

**Análisis de imágenes ecográficas utilizando redes neurales profundas para distinguir entre tumores ováricos benignos y malignos: comparación con la evaluación subjetiva de expertos**

**Objetivos:**

Desarrollar y probar el desempeño del análisis de imágenes ecográficas computarizadas utilizando redes neurales profundas (RNP) para distinguir entre tumores ováricos benignos y malignos y comparar su precisión en el diagnóstico con la de la evaluación subjetiva (ES) por especialistas expertos en ecografía.

**Métodos:**

Se incluyeron 3077 (escala de grises, n=1927; power Doppler, n=1150) imágenes de ultrasonido de 758 mujeres con tumores ováricos, que fueron clasificadas prospectivamente por examinadores especialistas en ecografía, de acuerdo con los términos y definiciones de la IOTA (Análisis Internacional de Tumores Ováricos). El resultado histológico de la cirugía (n=634) o el seguimiento a largo plazo (≥3 años) (n=124) sirvieron como el estándar de referencia. El conjunto de datos se dividió en un subconjunto de formación (n=508; 314 benignos y 194 malignos), un subconjunto de validación (n=100; 60 benignos y 40 malignos) y un subconjunto de pruebas (n=150; 75 benignos y 75 malignos). Se utilizó el aprendizaje de transferencia en tres RNP pre‐formadas: VGG16, ResNet50 y MobileNet. Cada modelo fue formado primero mediante escalas de temperatura, al igual que los la calibración de los *outputs*. A continuación, se utilizó una combinación de los tres modelos para estimar la probabilidad de que el tumor fuera maligno con base en la totalidad de las imágenes de un caso determinado. La combinación de RNP permitió clasificar los tumores como benignos o malignos (modelo Ovry‐Dx1); o como benignos, no concluyentes o malignos (modelo Ovry‐Dx2). Se comparó el desempeño para el diagnóstico de los modelos de RNP, en términos de sensibilidad y de especificidad, con el de la ES para la clasificación de los tumores ováricos en el subconjunto de formación.

**Resultados:**

Con una sensibilidad del 96,0%, Ovry‐Dx1 tuvo una especificidad similar a la de la ES (86,7% frente a 88,0%; P=1,0). Ovry‐Dx2 tuvo una sensibilidad del 97,1% y una especificidad del 93,7%, y designaron un 12,7% de las lesiones como no concluyentes. Cuando se complementó Ovry‐Dx2 con ES en los casos no concluyentes, la sensibilidad general (96,0%) y la especificidad (89,3%) no fueron significativamente diferentes de la utilización de ES en todos los casos (P=1,0).

**Conclusión:**

El análisis de imágenes ecográficas mediante RNP puede predecir el cáncer de ovario con una precisión en el diagnóstico igual a la de los especialistas expertos humanos, lo que indica que estos modelos pueden jugar un papel en el triaje de mujeres con un tumor de ovario. © 2020 The Authors. Ultrasound in Obstetrics & Gynecology published by John Wiley & Sons Ltd on behalf of International Society of Ultrasound in Obstetrics and Gynecology.

## 摘要

**采用基于深度神经网络的超声图像分析来辨别良性和恶性卵巢肿瘤:与专家主观评价对比**

**目的:**

开发并检测采用基于深度神经网络(DNN)的电脑化超声图像分析来辨别良性和恶性卵巢肿瘤的性能，并将其诊断准确性与由一名超声专家进行主观评价(SA)的诊断准确性进行对比。

**方法:**

我们包含了3077张(灰度图，n=1927；能量多普勒，n=1150)来自758名有卵巢肿瘤妇女的超声图像，她们已由专家超声检查人员根据IOTA(国际卵巢肿瘤分析)术语和定义进行预期分类。以手术的组织学预后(n=634)或长期(≥3年)随访(n=124)作为金标准。数据集被分成训练集(n=508；314个良性和194个恶性)、验证集(n=100；60个良性和40个L恶性)和测试集(n=150；75个良性和75个恶性)。我们将迁移学习用于三个预先训练的DNN:VGG16、ResNet50和MobileNet。每个模型都受过训练，对结果进行过标准化，采用温度定标。然后，根据给定病例的所有图像，三个模型整体被用于判断恶性的可能性。DNN整体将肿瘤分类为良性或恶性(Ovry‐Dx1模型)；或良性、不确定或恶性(Ovry‐Dx2模型)。从敏感性和特异性角度，就测试集中卵巢肿瘤的分类将DNN模型的诊断性能与SA的诊断性能进行对比。

**结果:**

当敏感度为96.0%时，Ovry‐Dx1的特异性类似于SA的特异性(86.7% vs 88.0%；P=1.0)。

在将12.7%的病灶指定为不确定时，Ovry‐Dx2的敏感度为97.1%，特异性为93.7%。在不确定的病例中用Ovry‐Dx2来补充SA，总体敏感度(96.0%)和特异性(89.3%)与在所有病例中使用SA(P=1.0)无显著差异。

**结论:**

基于DNN的超声图像分析可以用来预测卵巢恶性肿瘤，其诊断准确性能够达到人类专家检查人员的程度，表明这些模型可能在卵巢肿瘤妇女的分诊中起到作用。© 2020 作者。威利父子公司(John Wiley & Sons Ltd)代表国际妇产科超声学会(ISUOG)出版《国际妇产超声杂志》(Ultrasound in Obstetrics & Gynecology)。

**CONTRIBUTION**

**What are the novel findings of this work?**

Computerized ultrasound image analysis using deep neural networks (DNNs) can discriminate between benign and malignant ovarian lesions with a diagnostic accuracy comparable to that of a human expert examiner.

**What are the clinical implications of this work?**

There is a shortage of expert ultrasound examiners, resulting in suboptimal diagnostic accuracy. DNN models may have a role in the triage of women with an ovarian tumor, by supporting clinical decision‐making by less experienced examiners, and potentially reducing morbidity and optimizing the use of healthcare resources.

## Introduction

Ovarian cancer is the most lethal gynecological malignancy, with a global 5‐year survival of 45%^1^. Almost 10% of asymptomatic postmenopausal women have an ovarian lesion, often detected incidentally, of which only 1% are malignant^2^. Over 50% of ovarian tumors occur in fertile women^3^, in whom unnecessary or extensive surgery may cause fertility loss. Thus, there is a need to determine precisely the risk of malignancy to individualize and optimize treatment. Benign masses can be managed conservatively with ultrasound follow‐up or minimal invasive laparoscopy, while preserving fertility^3^. Women with suspected ovarian cancer should be referred directly to a gyneoncology treatment center, as surgical treatment of such patients by gynecological oncologists is associated with higher likelihood of complete tumor removal and improved survival rate^4^.

Expert ultrasound examination has become the main imaging technique for assessing ovarian lesions^5^. The diagnostic accuracy of ultrasound is better in the hands of experts than in the hands of less experienced doctors^6^, however, there is a shortage of expert examiners. Recent advances in computerized diagnostics have been powered by deep neural networks (DNNs), a class of machine‐learning algorithms that can learn complex representations of data from compositions of many simple non‐linear units. This approach is a paradigm shift, in which the input to the model is not hand‐designed, as in the past, but raw data^7^. DNNs have been shown to be able to discriminate between benign and malignant tumors in other domains, such as computed tomography (lung cancer)^8^, photographic imagery (skin cancer)^9^ and mammography (breast cancer)^10^, with performance on a par with that of expert radiologists. Using ultrasound images, DNNs have shown promising results in diagnosing breast and thyroid tumors^11^, ^12^, ^13^, although the field is still unexplored when it comes to ovarian tumors. Training DNNs requires large volumes of labelled data, which is often a scarce resource in the medical field. To overcome this limitation, transfer learning from ImageNet^14^, a large labelled dataset of roughly 1 million natural images, has become a standard practice for deep learning in medical applications^15^.

The aims of this study were to develop and test a DNN‐based computerized ultrasound image analysis model for discriminating between benign and malignant ovarian tumors and to compare its diagnostic accuracy with that of expert subjective assessment (SA)^16^ and IOTA (International Ovarian Tumor Analysis) simple rules^17^, ^18^ and simple‐rules risk^19^.

## Methods

### Dataset

We included retrospectively 3077 (grayscale, *n* = 1927; power Doppler, *n* = 1150) ultrasound images from 758 women with ovarian lesions. All women had undergone structured expert ultrasound assessment prior to surgery, at the gynecological ultrasound departments of the Karolinska University Hospital (tertiary referral center) and Södersjukhuset (secondary/tertiary referral center) in Stockholm, Sweden, between 2010 and 2019. The examinations were performed by one of six examiners with substantial experience (7–23 years) in the assessment of adnexal lesions. All examiners were certified (having undergone both theoretical and practical assessment) as second‐opinion expert sonographers, i.e. expert examiners, by the Swedish Society of Obstetrics and Gynecology (SFOG). Every case was assessed by a single examiner. Ethical approval was obtained by the local ethics committee (DNR 2010/145, 2011/343).

Eligible criteria were surgery within 120 days after the ultrasound examination (*n* = 634) or ultrasound follow‐up for a minimum of 3 years or until resolution of the lesion (*n* = 124). None of the women undergoing follow‐up was diagnosed with a malignant lesion, and thus, they were presumed to have a benign diagnosis. At the time of examination, a standardized protocol was filled out, in which the tumors were classified as benign or malignant using expert SA, the perceived certainty in the assessment was reported (uncertain *vs* certainly/probably benign/malignant), and the tumors were classified according to the IOTA simple rules. The IOTA simple rules include five benign and five malignant criteria. A lesion is considered as benign or malignant if only benign or malignant features, respectively, are present, and is classified as inconclusive if both benign and malignant features are present or none of the features is observed. Inconclusive lesions (approximately one in four cases) require a second‐stage test, usually SA by an expert examiner. If SA is not available, these lesions are considered potentially malignant^17^, ^18^. In order to be able to classify all lesions based on the simple‐rules assessment, we applied retrospectively the IOTA logistic regression model simple‐rules risk (SRR) to obtain a risk score for every case^19^. We used a cut‐off of 0.2 for SRR to define women with suspected malignancy, based on the original study^19^.

All examinations were performed using high‐end ultrasound systems, namely GE Voluson E8 or E10 with a 5–9‐ or 6–12‐MHz transducer (GE Healthcare, Zipf, Austria) or Philips IU22 or EPIQ with a 3–10‐MHz transducer (Philips Medical Systems, Bothell, WA, USA). Eligible cases were selected randomly from our ovarian tumor database. Benign and malignant cases were selected separately, to obtain a case‐mix with at least 40% malignant cases (as the clinical incidence of malignancy among women undergoing expert ultrasound assessment prior to surgery was 40% at Södersjukhuset and 49% at Karolinska University Hospital during the study period). In the selected cases, representative grayscale and power Doppler ultrasound images (median, 3 (interquartile range, 3–5) images per case), in which the whole lesion was adequately shown, were selected and downloaded in jpeg format from the hospital image database systems. The images were deidentified and then cropped manually by the reviewer to the region of interest (ROI), which in the vast majority of images involved removing merely the outer borders and occasionally also excluding surrounding structures, such as the uterus (Figure S1).

Following standard practice, our dataset was split into a training set (*n* = 508; 314 benign and 194 malignant), a validation set (*n* = 100; 60 benign and 40 malignant) and a test set (*n* = 150; 75 benign and 75 malignant, with three images per case). The training set was used to learn the parameters of the models; the validation set was used to estimate the prediction error for hyperparameter tuning and model selection; and the test set was used to assess independently the generalization error for the final chosen models, preventing possible overfit to the training data. To ensure accurate results, only patients with a histological diagnosis obtained by surgery were included in the test set. The histological outcome for all women who underwent surgery, and separately for those who were included in the test set, is shown in Table 1.

**Table 1:** Histological outcome of all women with ovarian lesions who underwent surgery and separately for subset included in test set

Histological outcome	All cases (*n* = 634)	Test set (*n* = 150)
Benign	325 (51.3)	75 (50.0)
Endometrioma	46 (7.3)	10 (6.7)
Dermoid	74 (11.7)	26 (17.3)
Simple/functional cyst	31 (4.9)	3 (2.0)
Paraovarian cyst	12 (1.9)	—
Rare benign	9 (1.4)	1 (0.7)
(Hydro‐)pyosalpinx	14 (2.2)	3 (2.0)
Fibroma/myoma	25 (3.9)	5 (3.3)
Cystadenoma/cystadenofibroma	108 (17.0)	25 (16.7)
Peritoneal/inclusion cyst	6 (0.9)	2 (1.3)
Borderline	55 (8.7)	15 (10.0)
Serous	35 (5.5)	8 (5.3)
Mucinous	20 (3.2)	7 (4.7)
Malignant	254 (40.1)	60 (40.0)
Epithelial ovarian cancer	169 (26.7)	38 (25.3)
Non‐epithelial ovarian cancer	28 (4.4)	10 (6.7)
Metastatic ovarian tumor	57 (9.0)	12 (8.0)

Data are presented as *n* (%).

### Data processing

Images with a shape of (224, 224, 3), i.e. square images measuring 224 by 224 pixels with three color channels (RGB), were used as input to the DNN models. Therefore, the images were downsampled and resized accordingly, using nearest‐neighbor interpolation. Since the images were not at a uniform scale, the resulting physical resolution varied. Furthermore, the pixel values were standardized channel‐wise to have zero mean and unit variance over the training dataset. The same values were also used to standardize the validation and test sets.

Data augmentation was performed during training for model generalization by expanding the available training data and mimicking shifts in image properties in unseen domains. The transformations used in the augmentation process can be divided into three main categories^20^, based on the aspect of the image that is altered: image quality, spatiality and appearance. Low image quality is characterized mainly by blurriness and low resolution, caused by scanner motion, low scanner resolution or lossy image compression. We tried to imitate this by adding Gaussian noise (std, 0.02), jpeg compression (0–20%) and shift in sharpness (± 20%). Transformations related to the spatial shape of the images comprised horizontal and vertical flips, rotation (multiples of 90°) and cropping (0–10%). These transformations served the purpose of simulating the variability in the shape and position of organs, the size of patients and ROI‐related cropping. The appearance‐related transformations were shifts in brightness (± 20%), contrast (± 20%) and color (± 10%), which mimicked the differences between ultrasound systems and settings.

### Model building

We used a transfer‐learning approach on ImageNet^14^ pre‐trained deep‐learning models VGG16^21^, ResNet50^22^ and MobileNet^23^. Each model was fine‐tuned on the dataset of transvaginal ultrasound images of ovarian tumors. The output probabilities were then calibrated independently using temperature scaling^24^, a post‐processing technique used to better align the confidence scores with the underlying class probabilities (Figure S2). The three models were then combined into an ensemble, using a soft voting scheme of averaging the probabilities from the models, to improve performance over the individual models. Temperature scaling ensured more reliable estimates of the probability of malignancy for each model, which is desirable when building an ensemble, as it reduces the problem of difference in (over‐) confidence between the models. The ensemble was then used to estimate the probability of malignancy of a given case, by averaging the predictions from all representative images from that case. Using the prediction from the ensemble, tumors were classified as benign or malignant (Ovry‐Dx1) or as benign, inconclusive or malignant (Ovry‐Dx2), by setting thresholds on the predicted probability of malignancy. The performance of the DNN models was compared to that of SA, based on their sensitivity and specificity in discriminating between benign and malignant lesions in the test set. The probability threshold for Ovry‐Dx1 was intended to be set so as to give an optimal balance between sensitivity and specificity, with a sensitivity close to that of SA. Since this value was near 0.5 and its uncertainty large, the threshold was simply set to 0.5, with cases above this threshold classified as malignant. For Ovry‐Dx2, the probability thresholds were set to 0.4 and 0.6 (i.e. cases with a predicted probability of malignancy between 0.4 and 0.6 were classified as inconclusive), which was shown to result in a reasonable balance between performance and fraction of excluded cases in the validation set.

### Training process

First, in each model, the original classifier for the ImageNet classes was replaced by a binary classifier, consisting of a fully connected layer of 1024 hidden nodes (512 for MobileNet), followed by ReLU‐activation, dropout of 0.5 (0.2 for VGG16) and a final fully connected softmax layer with two nodes representing the benign and malignant outputs. In the first training step, the weights of the convolutional base of the original models were frozen, thereby training only the new binary classifier, using an initial learning rate of 0.02 (0.002 for VGG16). Then, the layers of the convolutional base were unfrozen and fine‐tuned one by one, until no more improvement could be seen. An initial learning rate of 2 × 10^− 4^ was used in this step. All models were trained using backpropagation^25^ by stochastic gradient descent, with Nesterov momentum^26^ of 0.9 and a batch size of 32 images, on an NVIDIA RTX 2080 graphics card. Imbalance in the number of grayscale and power Doppler images, from benign and malignant cases, was addressed by training with weighted binary cross‐entropy loss. We used a learning‐rate decay of 0.5 after every four consecutive epochs of no improvement in validation accuracy. In both steps of training, we used early stopping to monitor the performance of the model on the validation set and select the optimal point to stop training (Figure S3). This is the point at which the model performance on the validation set stops improving, and any additional training overfits the training data at the expense of increased generalization error. The hyperparameters, such as the learning rate, dropout rate and the number of hidden nodes, were chosen to maximize performance on the validation set.

Similar to most modern deep‐learning architectures, ResNet50 and MobileNet use batch normalization^27^ to improve training performance and stability. When using transfer learning on a model with batch normalization layers, the difference between the source domain (natural images) and target domain (ultrasound images) must be addressed. If the parameters of these layers are frozen during training of the new classifier, the model will be using the exponential moving average (EMA) statistics from the source domain for normalization at test time. These statistics will differ significantly from the statistics of the batches during training of the new classifier in the target domain, leading potentially to a large drop in performance. To align the training and test behavior of the model, the EMA statistics of the batch normalization layers were updated continually during fine‐tuning of the models in the target domain.

### Evaluation of possible image bias

Caliper measurements were present in ∼80% of both benign and malignant images in the dataset. To rule out any potential bias introduced by the calipers, the final ensemble model (Ovry‐Dx1) was evaluated on images with and without calipers. Since we were not interested in the absolute, but rather the relative performance, we used both the validation and test datasets to obtain a more accurate measurement of the effect. Furthermore, only the grayscale images were used, since very few power Doppler images contained calipers. On the images with and without calipers, the sensitivities (88.8% *vs* 89.6%) and specificities (79.1% *vs* 82.4%) of Ovry‐Dx1 were similar, and the outputs of the network were statistically indistinguishable (*P* = 0.86 and *P* = 0.50). This indicates that the presence of caliper measurements does not play a significant role in assisting the model in predicting malignancy of ovarian tumors.

To rule out potential bias related to the use of power Doppler images in addition to using only grayscale images, the final ensemble model (Ovry‐Dx1) was evaluated on the test set for each individual image alone. On the power Doppler and grayscale images, the specificities (85.7% *vs* 85.1%) were similar, while the sensitivity was higher for power Doppler images compared to grayscale images (88.2% *vs* 82.9%). Furthermore, the outputs of the network on the benign images were statistically indistinguishable (*P* = 0.61), while the outputs of the network on the malignant images were statistically higher for the power Doppler images (*P* = 0.03). Based on this, the model's response to benign images was similar for both modalities. There does appear to be a slight difference in the network's response towards malignant images in favor of the power Doppler modality, but the higher sensitivity may be explained by the addition of blood flow information. In practice, this means that cases with power Doppler images, in addition to grayscale images, will likely see an increase in sensitivity without a drop in specificity.

### Statistical analysis

To compare the performance of the DNN models to that of SA in discriminating between benign and malignant tumors in the test set, the sensitivity, specificity, accuracy and area under the receiver‐operating‐characteristics (ROC) curve (AUC), with their 95% CI, were calculated. The 95% CI for the sensitivity, specificity and accuracy where estimated by the Jeffreys interval^28^, while the 95% CI for the AUC and the fraction of excluded cases were estimated by bootstrapping. Comparison of the sensitivity, specificity and accuracy of the DNN models with that of SA, simple rules and SRR was performed using McNemar's test for paired categorical data. Evaluation of possible image bias, from caliper measurements and power Doppler images, was based on the Mann–Whitney *U*‐test for unpaired non‐parametric data. Statistical analysis was performed using IBM SPSS Statistics for Windows, version 26.0 (IBM Corp., Armonk, NY, USA). All tests were two‐sided and *P*‐values < 0.05 were considered statistically significant.

**Figure 1:**
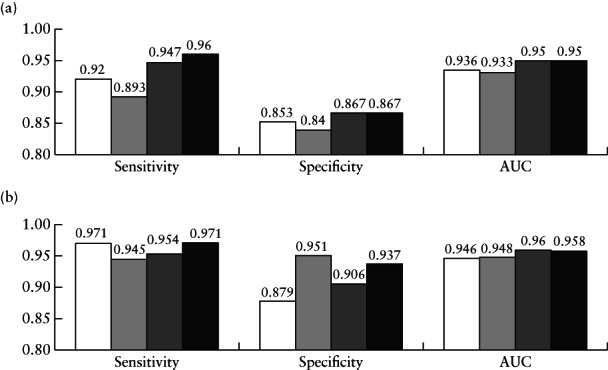
Diagnostic performance for distinguishing between benign and malignant ovarian tumors of deep‐learning models VGG16 (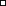), ResNet50 (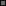), MobileNet (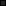) and ensemble of the three models (Ovry‐Dx1 in (a) and Ovry‐Dx2 in (b); (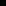)), in all patients in test set (a) and when excluding cases with predicted probability of malignancy between 0.4 and 0.6, corresponding to high uncertainty (b). Percentage of cases excluded was 10.7% for VGG16, 22.7% for ResNet50, 14.0% for MobileNet and 12.7% for Ovry‐Dx2. AUC, area under receiver‐operating‐characteristics curve.

**Figure 2:**
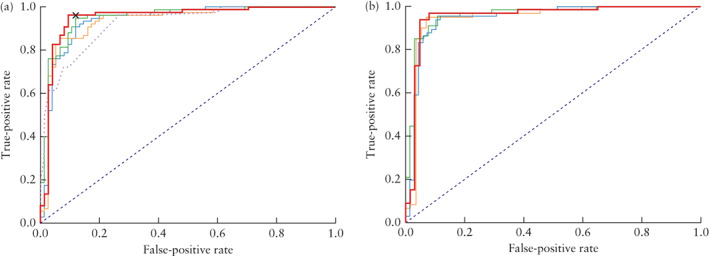
Receiver‐operating‐characteristics (ROC) curves for distinguishing between benign and malignant ovarian tumors of deep‐learning models VGG16 (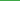), ResNet50 (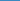) and MobileNet (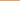) and ensemble of the three models (Ovry‐Dx1 in (a) and Ovry‐Dx2 in (b); (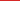)), in all patients in test set (a) and when excluding inconclusive cases (predicted probability of malignancy between 0.4 and 0.6) (b). In (a), ROC curve of IOTA simple‐rules risk (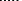) and operating point for expert subjective assessment (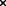) are also shown.

## Results

The performance statistics on the test set of the three DNN models (VGG16, ResNet50 and MobileNet) and the ensemble of these models are shown in Figure 1 and the corresponding ROC curves in Figure 2. The ensemble model was the overall best performing model, achieving an AUC of 0.950 (95% CI, 0.906–0.985) when all cases in the test set were classified as benign or malignant (Ovry‐Dx1), and an AUC of 0.958 (95% CI, 0.911–0.993) when excluding 12.7% (95% CI, 7.3–18.0%) of cases as inconclusive (Ovry‐Dx2). In Table 2, we present the diagnostic performance of SA, simple rules, SRR and the DNN models (Ovry‐Dx1 and Ovry‐Dx2) for differentiating between benign and malignant ovarian lesions in the test set. At a sensitivity of 96.0%, Ovry‐Dx1 had a specificity similar to that of SA (86.7% *vs* 88.0%; *P* = 1.0). Ovry‐Dx2 had a sensitivity of 97.1% and a specificity of 93.7%, when designating 12.7% (95% CI, 7.3–18.0%) of the lesions as inconclusive. By complementing Ovry‐Dx2 with SA in inconclusive cases, the overall sensitivity (96.0%) and specificity (89.3%) were not significantly different from using SA in all cases (*P* = 1.0). Use of the simple rules classified 27.3% of cases as inconclusive. At a sensitivity of 96.0%, Ovry‐Dx1 had a significantly higher specificity (86.7% *vs* 66.7%; *P* = 0.003) and accuracy (91.3% *vs* 81.3%; *P* = 0.006) than the simple rules with inconclusive cases classified as malignant (Table 2). At a cut‐off of 0.2, SRR had the same performance as the simple rules with inconclusive cases classified as malignant (Table 2). The cut‐off of 0.2 was chosen based on the results of the original study^19^, and furthermore, any cut‐off between 0.153 and 0.325 would have resulted in the same performance, since no case was given a prediction in this interval. SRR achieved an AUC of 0.921 (95% CI, 0.876–0.958).

**Table 2:** Diagnostic performance of expert subjective assessment (SA)^16^, IOTA simple rules (SR)^17^, ^18^, IOTA simple‐rules risk (SRR)^19^ and deep neural network models Ovry‐Dx1 and Ovry‐Dx2, for discriminating between benign and malignant ovarian lesions in test set (*n* = 150)

		Sensitivity			Specificity			Accuracy		
Diagnostic model	Percent that could be classified	*n*/*N*	% (95% CI)	*P*	*n*/*N*	% (95% CI)	*P*	*n*/*N*	% (95% CI)	*P*
SA in all cases	100	72/75	96.0 (89.7–98.9)	N/A	66/75	88.0 (79.2–93.9)	N/A	138/150	92.0 (86.8–95.6)	N/A
Ovry‐Dx1 in all cases	100	72/75	96.0 (89.7–98.9)	1.0‡	65/75	86.7 (77.6–92.9)	1.0‡	137/150	91.3 (86.0–95.1)	1.0‡
Ovry‐Dx2 excluding inconclusive cases	87.3	66/68	97.1 (90.9–99.4)	N/A	59/63	93.7 (85.6–97.8)	N/A	125/131	95.4 (90.8–98.1)	N/A
Ovry‐Dx2 + SA in inconclusive cases	100	72/75	96.0 (89.7–98.9)	1.0‡	67/75	89.3 (80.9–94.8)	1.0‡	139/150	92.7 (87.7–96.0)	0.75‡
SR only	72.6	52/55	94.5 (86.2–98.4)	N/A	50/54	92.6 (83.3–97.4)	N/A	102/109	93.6 (87.8–97.1)	N/A
SR with inconclusive cases as malignant*	100	72/75	96.0 (89.7–98.9)	1.0§	50/75	66.7 (55.5–76.5)	0.003§	122/150	81.3 (74.5–86.9)	0.006§
SR + SA in inconclusive cases	100	71/75	94.7 (87.8–98.2)	1.0§	65/75	86.7 (77.6–92.9)	1.0§	136/150	90.7 (85.2–94.5)	1.0§
SRR in all cases†	100	72/75	96.0 (89.7–98.9)	1.0§	50/75	66.7 (55.5–76.5)	0.003§	122/150	81.3 (74.5–86.9)	0.006§

* Cases in which SR yielded inconclusive result were classified as malignant.

† Using cut‐off of 0.2 to define women with suspected malignancy. McNemar's test used for comparison of:

‡ Ovry‐Dx1 and Ovry‐Dx2 to SA; §Ovry‐Dx1 to SR or SRR. N/A, not applicable.

**Figure 3:**
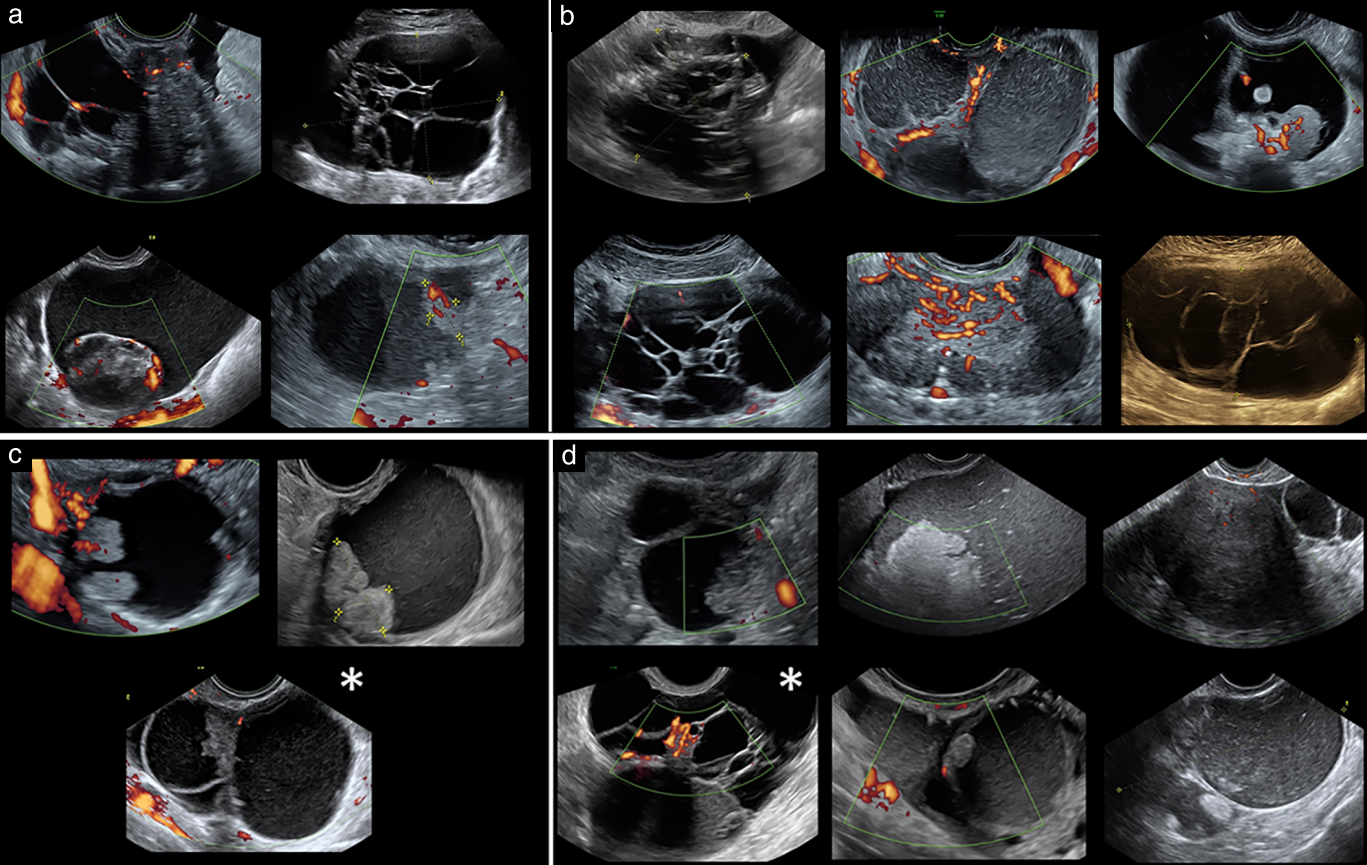
(a,b) Ten ovarian lesions that were difficult to classify by both expert subjective assessment and deep neural network model Ovry‐Dx2; final histological diagnosis was malignant or borderline in four (a) and benign in six (b). (c,d) Further nine ovarian lesions classified as inconclusive by Ovry‐Dx2; final histological diagnosis was malignant or borderline in three (c) and benign in six (d). Images marked with (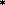) were also classified as inconclusive by IOTA simple rules.

**Figure 4:**
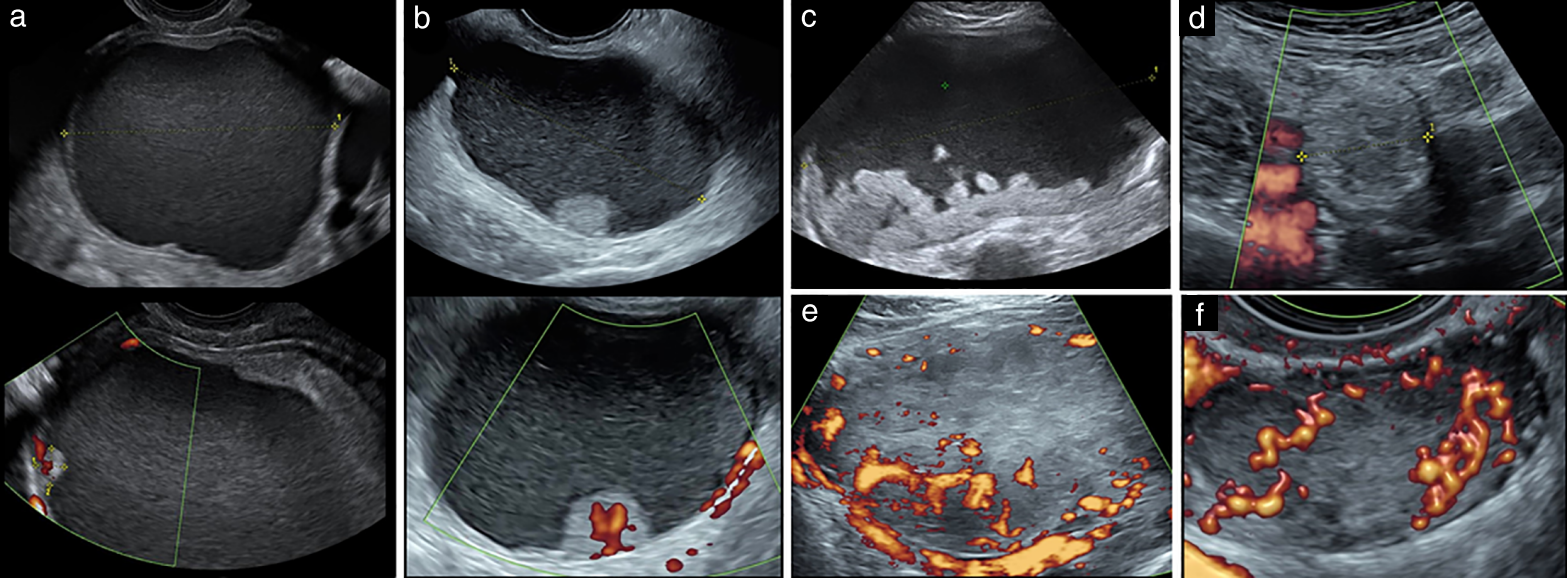
Six cases misdiagnosed by deep neural network model Ovry‐Dx2: (a,b) malignant cases classified as benign; (c–f) benign cases classified as malignant. (a) Clear‐cell carcinoma with 6‐mm papillary projection; case was also misdiagnosed by expert subjective assessment. (b) Mucinous intestinal borderline tumor. (c,d) Dermoid. (e) Fibroma; case was also misdiagnosed by expert subjective assessment. (f) Leydig cell tumor.

Table 3 shows the histological outcome of cases classified as inconclusive by Ovry‐Dx2 and simple rules and cases that were difficult to classify by SA. Of the 19 cases classified as inconclusive by Ovry‐Dx2, 10 were also considered difficult to classify by SA (Table 2, Figure 3a,b). Figure 3c,d shows the other nine cases that were classified as inconclusive by Ovry‐Dx2, of which two were also considered inconclusive by simple rules. Figure 4 shows the six cases that were misdiagnosed by Ovry‐Dx2, two of which were also misdiagnosed by SA.

**Table 3:** Histological outcome in ovarian lesions that were inconclusive by IOTA simple rules and deep neural network model Ovry‐Dx2, and difficult to classify by expert subjective assessment (SA)

Histological outcome	SA uncertain (*n* = 27)	Simple rules inconclusive (*n* = 41)	Ovry‐Dx2 inconclusive (*n* = 19)	Overlapping inconclusive/uncertain cases by Ovry‐Dx2 and SA (*n* = 10)
Benign				
Endometrioma	1	2	1	—
Dermoid	5	3	4	2
Simple cyst	—	1	—	—
Paraovarian	—	—	—	—
Rare benign	1	—	—	—
(Hydro‐)pyosalpinx	—	—	—	—
Fibroma/myoma	1	2	2	1
Cystadenoma/cystadenofibroma	9	13	5	3
Peritoneal/inclusion cyst	1	—	—	—
Borderline malignant				
Serous	2	2	2	1
Mucinous	3	5	2	2
Invasive malignant				
Epithelial ovarian cancer	3	7	3	1
Non‐epithelial ovarian cancer	1	3	—	—
Metastatic ovarian tumor	—	3	—	—

Data are given as *n*.

## Discussion

We have shown that ultrasound image analysis using DNNs can predict ovarian malignancy with a diagnostic accuracy comparable to that of human expert examiners. Furthermore, we found a substantial overlap between lesions difficult to classify by SA and by Ovry‐Dx2, indicating that both the expert and the DNNs recognize features (e.g. papillary projections, multilocular lesions with > 10 loculi) known to signify diagnostic difficulties^29^.

While our work is unprecedented in applying deep learning for classification of adnexal masses, a few pilot studies with a limited number of cases have explored the use of handcrafted image descriptors, in combination with support vector machines (SVM), for classification. Two studies^30^, ^31^ on 177 patients used local binary patterns (LBP)^32^ and pixel intensity histograms as texture features; the first reported an accuracy of 76%, while excluding 18.3% of the cases as inconclusive^30^, and the second a sensitivity of 77% and specificity of 77%^31^, for classifying ovarian masses. A later study^33^ on the same dataset achieved an increased performance (sensitivity 91%, specificity 83%, AUC of 0.874) using Fourier transform‐based feature descriptors and SVM for classification. While the latter results are promising, these studies all share the important limitation that they relied on manual segmentation of tumors by an ultrasound expert. The selection of a rectangular ROI in our own work is of an entirely different nature, as it requires significantly less involvement and domain expertise by the operator. While we identified other related papers^34^, we have omitted these due to methodological shortcomings, e.g. reporting classification performance on the same data used for selection of image features. Finally, the key to the success of DNNs is their ability to learn highly representative features, on multiple scales and levels of abstraction, directly from large datasets of raw images. This leads to features of greater discriminative capacity compared to conventional handcrafted descriptors^35^.

An advantage of our model is that it is simple to use, as any center could upload a set of deidentified images directly from the workstation or hospital computer, to a cloud platform hosting the model, without the need to first assess subjectively the images or provide additional patient data. The greatest clinical benefit of a diagnostic DNN model for classification of ovarian tumors would be in the hands of non‐expert examiners; however, it may also be useful to experts as a second reader. Many centers and private practitioners have limited access to a second opinion by an ultrasound expert; therefore, they might use simple rules, designating inconclusive cases as malignant, or use SRR in order to reach an acceptable sensitivity and be able to manage all patients. In this setting, the specificity could potentially be improved by 20 percentage points (from 66.7% for both simple rules strategies, to 86.7%) by instead using Ovry‐Dx1 in all cases (Table 2). In addition to high sensitivity, a high specificity is also of utmost importance, as many lesions are detected incidentally in asymptomatic women. Unnecessary surgery misuses the resources of the healthcare system and may cause morbidity, and in some cases even fertility loss. In a large randomized screening study for ovarian cancer^36^, one‐third of women with false‐positive findings underwent adnexal surgery. Of these, 15% experienced at least one major complication, highlighting the importance of minimizing false‐positive diagnoses in an asymptomatic population. By adjusting thresholds on the DNN predictions, as we have done for Ovry‐Dx2, it is possible to optimize sensitivity, specificity and the fraction of inconclusive cases, depending on the setting (i.e. low‐ or high‐resource) and the population (i.e. low or high risk of disease).

A strength of this study is that all cases were classified prospectively by expert ultrasound examination prior to surgery or long‐term ultrasound follow‐up, which enabled us to compare the performance of the DNNs to that of SA. The proportion of cases classified as inconclusive by simple rules (27.3%) is slightly higher than that reported in previous studies (22.5%^17^ and 24.0%^18^), indicating that the number of difficult cases in our test set is similar to or higher than that in other populations. While no direct comparison can be made, the sensitivity (96.0%) and specificity (88.0%) of SA in our test set, are comparable to the reported performance in a large IOTA multicenter study including 1938 cases (sensitivity 90.4%, specificity 92.7%)^17^.

We included a wide range of ovarian pathology with high‐quality images, optimizing DNN model development. However, homogeneity of the image quality can be seen as a weakness of this study, as the majority of images were obtained by the same examiner using the GE Voluson E8 or E10 systems. Thus, it remains to be shown if the models perform equally well on images acquired by other expert centers, less experienced examiners or by examiners not using high‐end equipment. Evaluating cases based on batches of images led to better diagnostic accuracy compared to using single images. A next step could be to explore if the use of two‐dimensional videoclips or three‐dimensional volumes could further improve evaluation performance. Additional data, especially from diagnoses with low prevalence, could allow training of a DNN for multiclass diagnosis‐specific classification.

In this study, images were cropped manually, mainly by removing the outer borders; a task that would render itself suitable for auto‐cropping. As the selection of an acceptable ROI requires only the ability to locate the tumor in the recorded image, which is a requirement for recording the image in the first place, this coarse cropping does not depend on expertise beyond that which is already inevitable for image acquisition. Still, despite the fact that manual ROI selection is performed in most studies on deep learning in the medical domain^37^, the necessity of manual cropping and the potential benefit of auto‐cropping should be explored in future studies. Finally, our extensive spatial data augmentation during training is further likely to increase robustness and flexibility in the ROI selection.

We would like to emphasize the importance of external validation of the Ovry‐Dx1 and Ovry‐Dx2 models on images from other gynecological centers, in order to evaluate the limitation in generalization and the potential for multisite deployment. Thus, the next step is to validate externally these models in a large multicenter setting, which is already underway (ISRCTN51927471). It should be stressed that automated image analysis should only be used to assist in the triage of patients and not to make a final diagnosis. Nevertheless, our results clearly indicate that DNNs have the potential to be clinically useful in the triage of women with an ovarian tumor.

## Supporting information


**Figure S1** Cropping of ultrasound images to standardized dimensions of 4:3 by selecting region of interest (ROI).

**Figure S2** Reliability diagrams for VGG16‐based model, before and after calibration, showing accuracy plotted against confidence of the model. Confidence is the predicted probability for the most probable class (benign or malignant). Since classification is binary, it will always be above 0.5. In an overconfident model, confidence exceeds accuracy.

**Figure S3** Model accuracy during training for VGG16, ResNet50 and MobileNet models. Dashed lines indicate early stopping points used in final models.

## Data Availability

The data that support the findings of this study are available upon reasonable request from the corresponding author. The data are not publicly available due to privacy or ethical restrictions.
