# Supplementary figures and images for: Ultrasound image analysis using deep neural networks for discriminating between benign and malignant ovarian tumors: comparison with expert subjective assessment
Source: Ultrasound Obstet Gynecol. 2021 Jan 2;57(1):155–63. doi: 10.1002/uog.23530 (PMC7839489; doi:10.1002/uog.23530)

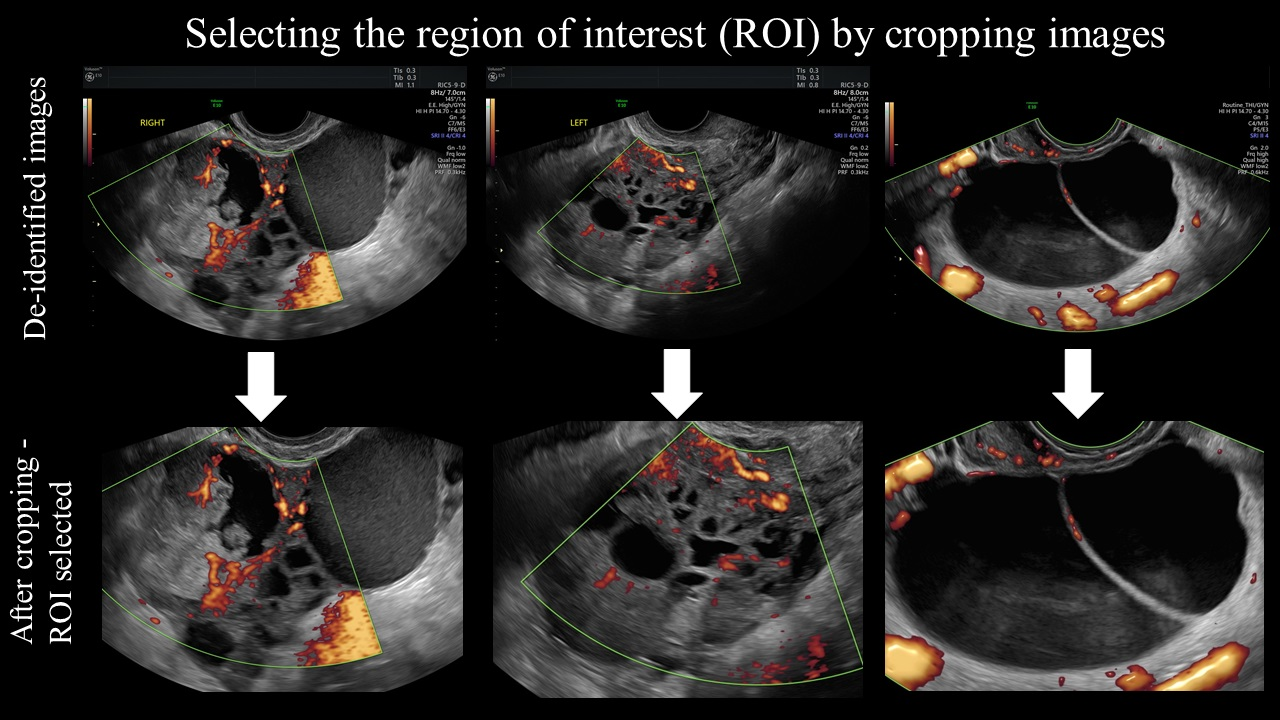

Supplement: Supplementary file 1 — Figure S1 Cropping of ultrasound images to standardized dimensions of 4:3 by selecting region of interest (ROI). [file UOG-57-155-s001.tif]

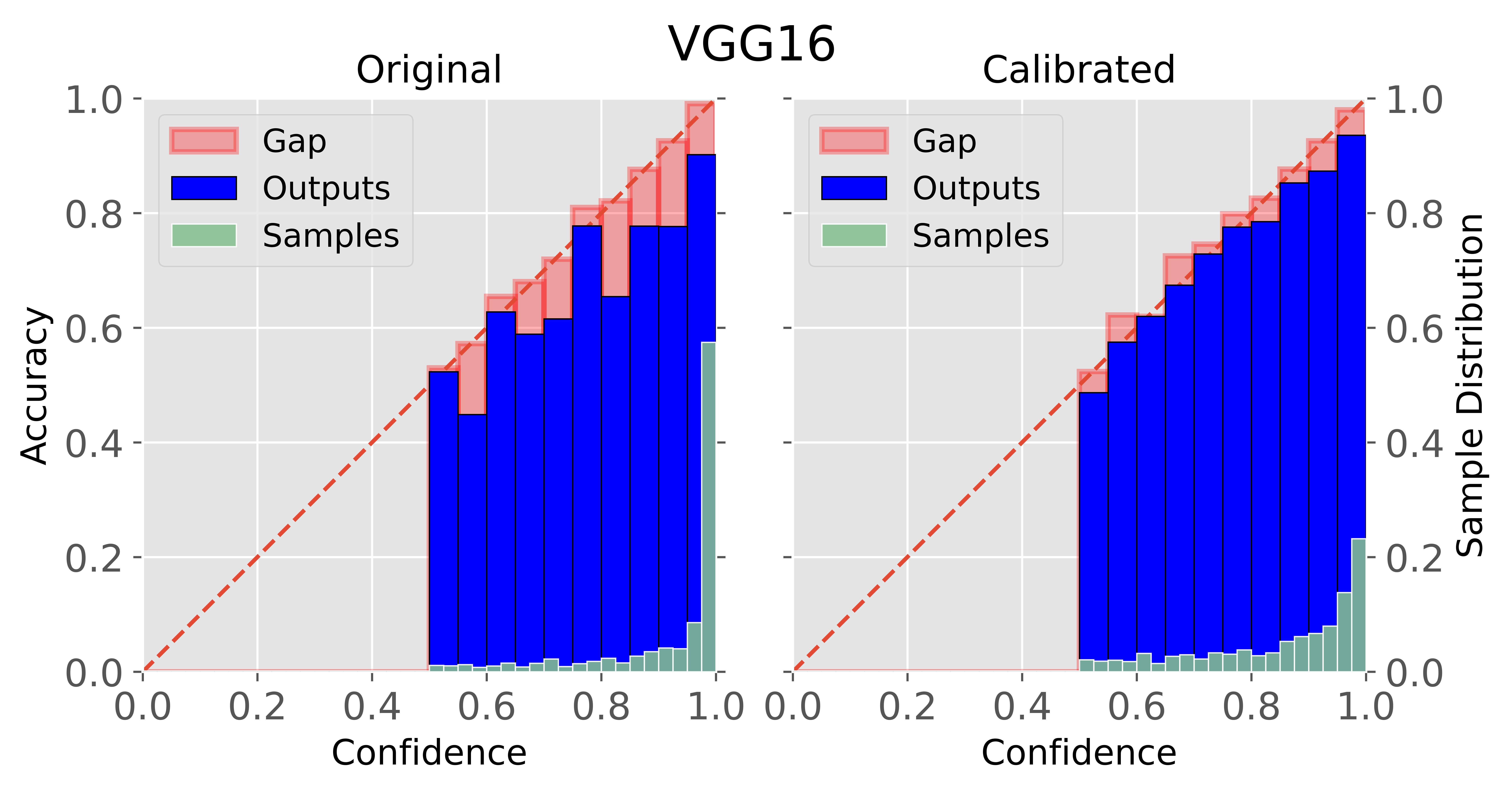

Supplement: Supplementary file 2 — Figure S2 Reliability diagrams for VGG16‐based model, before and after calibration, showing accuracy plotted against confidence of the model. Confidence is the predicted probability for the most probable class (benign or malignant). Since classification is binary, it will always be above 0.5. In an overconfident model, confidence exceeds accuracy. [file UOG-57-155-s002.tif]

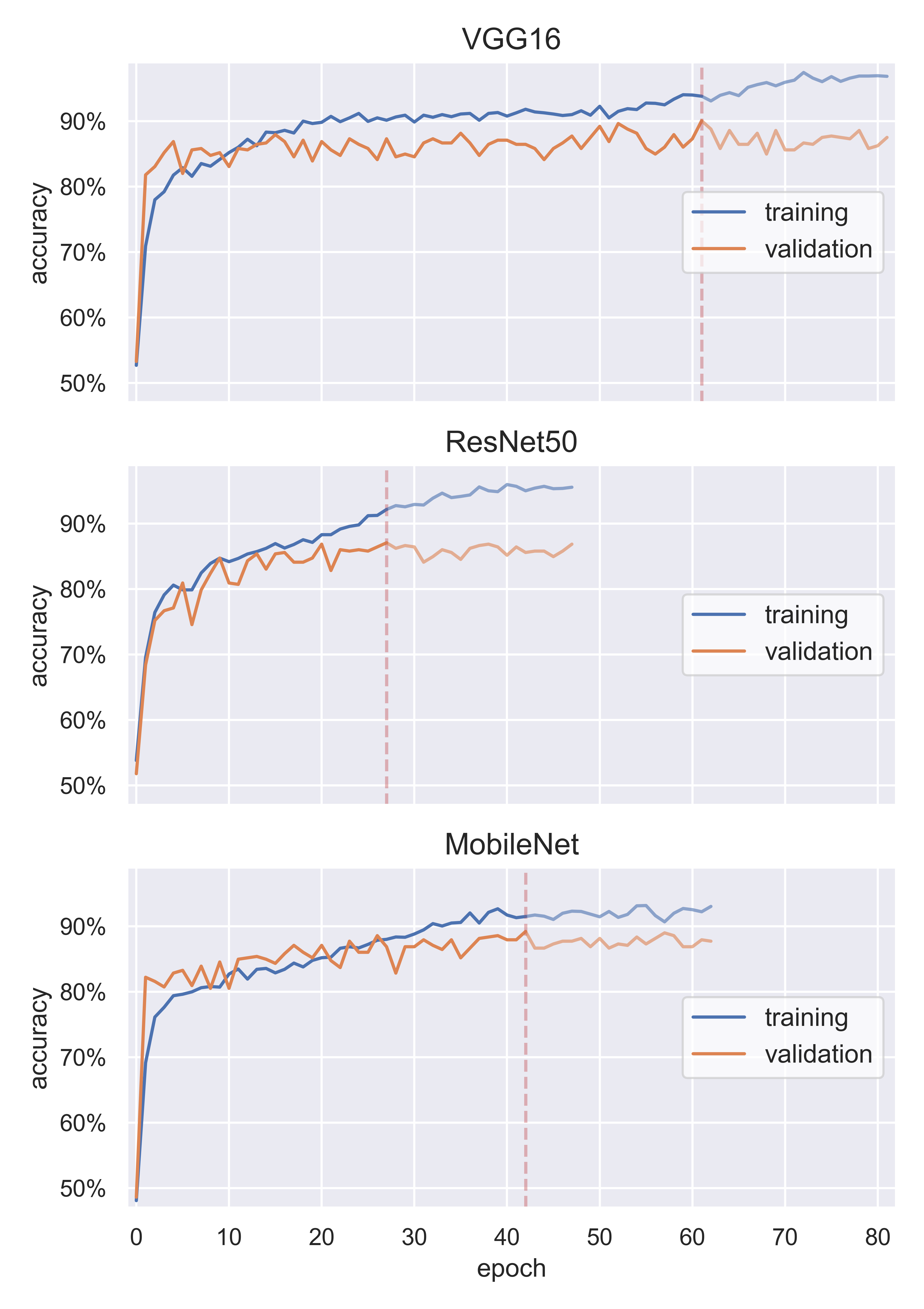

Supplement: Supplementary file 3 — Figure S3 Model accuracy during training for VGG16, ResNet50 and MobileNet models. Dashed lines indicate early stopping points used in final models. [file UOG-57-155-s003.tif]
